# Negative Peer Relationships on Piracy Behavior: A Cross-Sectional Study of the Associations between Cyberbullying Involvement and Digital Piracy

**DOI:** 10.3390/ijerph14101180

**Published:** 2017-10-05

**Authors:** Santiago Yubero, Elisa Larrañaga, Beatriz Villora, Raúl Navarro

**Affiliations:** Faculty of Education and Humanities, Department of Psychology, University of Castilla-La Mancha, Avda de los Alfares, 42, 16071 Cuenca, Spain; santiago.yubero@uclm.es (S.Y.); elisa.larranaga@uclm.es (E.L.); beatriz.villora@uclm.es (B.V.)

**Keywords:** cyberbullying, digital piracy, ethical behavior, adolescents, online violence

## Abstract

The present study examines the relationship between different roles in cyberbullying behaviors (cyberbullies, cybervictims, cyberbullies-victims, and uninvolved) and self-reported digital piracy. In a region of central Spain, 643 (49.3% females, 50.7% males) students (grades 7–10) completed a number of self-reported measures, including cyberbullying victimization and perpetration, self-reported digital piracy, ethical considerations of digital piracy, time spent on the Internet, and leisure activities related with digital content. The results of a series of hierarchical multiple regression models for the whole sample indicate that cyberbullies and cyberbullies-victims are associated with more reports of digital piracy. Subsequent hierarchical multiple regression analyses, done separately for males and females, indicate that the relationship between cyberbullying and self-reported digital piracy is sustained only for males. The ANCOVA analysis show that, after controlling for gender, self-reported digital piracy and time spent on the Internet, cyberbullies and cyberbullies-victims believe that digital piracy is a more ethically and morally acceptable behavior than victims and uninvolved adolescents believe. The results provide insight into the association between two deviant behaviors.

## 1. Introduction

Digital piracy is defined as the illegal act of copying or downloading digital goods, such as software, games, books, music, movies, and TV series, without obtaining the explicit permission from, and paying compensation to, the copyright holder [1,2]. According to the 2017 MUSO Global Piracy Report [3], in 2016 there were 191 billion visits to piracy sites worldwide. The year 2016 witnessed a sharp downturn in torrent usage, while streaming holds its ground as the most popular method that audiences choose to access illegal content, with 77.7 billion global visits. Mobile devices are used to perform 34% of piracy activity. Piracy is a form of criminal behavior that has infiltrated every country and annually costs the global economy many billions of dollars [4].

In 2016 in Spain, 4128 billion illegal accesses to content took place, valued at 23,294 billion euros, which causes a loss in profit valued at 1783 billion euros. Consumers who access illegal content claim that “digital contents are very expensive” or “I’m already paying for my Internet connection”, with 47% of users arguing that in both cases. Twenty-four percent of users also claim “I’m not doing anybody any harm” or “there are no legal consequences for those who pirate; nothing happens” [5].

Worldwide data show that digital piracy has slightly diminished in terms of absolute figures. However, the percentage of citizens who assess pirate content remains the same, or has even increased for some sectors, such as music and leisure books [3]. Consequently, the study of the social and individual drivers that guide digital piracy still warrants attention and inquiry.

### 1.1. Predictors of Digital Piracy

To effectively intervene to reduce the prevalence of digital piracy, research has been conducted to determine the predictors of digital piracy in youths. Self-efficacy to commit piracy and low self-regulation are the strongest predictors of digital piracy [6,7]. Viewing digital piracy as ethical behavior also seems to be a strong predictor of a person's intentions to pirate, and also of actual piracy behavior [8]. Indeed the existing literature has found that digital piracy offenders do not view piracy as being illegal or unethical [9,10,11]. Outcome expectations are also associated with digital piracy. Specifically, extrinsic rewards (i.e., saving money) and intrinsic rewards (i.e., fun, excitement) are more influential on piracy behaviors than risk (i.e., potential negative social and financial costs) and sanctions (i.e., the likelihood of prosecution) associated with piracy. Apart from the individual factors related with piracy, another important predictor is the peer’s negative influence (i.e., peer deviation, coercive pressure, pirating peers) associated with the odds of digital piracy being higher [10,12]. However, previous research has largely focused on personal variables and social norms. Less research has been conducted to examine the influence of negative peer relationships, specifically cyberbullying, on self-reported digital piracy in youths.

### 1.2. Cyberbullying

Cyberbullying refers to “willful and repeated harm inflicted through computers, cell phones, and other electronic devices” [13]. Researchers emphasize the electronic nature of aggressive behavior and focus on those behaviors that are deliberate, occur over time and result in harm. Cyberbullying is known as a global phenomenon that cuts across cultural groups and contexts [14]. According to a recent meta-analysis [15], the frequency of being involved in cyberbullying perpetration is about 16%, and is around 15% in cyberbullying victimization. Hamm et al. [16] reported median rates of cyberbullying perpetration and victimization of 15.2% and 23%, respectively. Survey data collected from among international youth populations have shown a drop in general peer victimization in the last decade, while online harassment has steadily increased since 2000 [17]. However, cyberbullying remains far less prevalent, with rates less than half those of traditional bullying [18].

Cyberbullying significantly affects children’ and adolescents’ social, emotional, and academic well-being. Different studies have shown that victims of cyberbullying are more likely than non-victimized youths to report emotional distress, depression symptoms, low self-esteem, anxiety, social isolation, less life satisfaction, school absenteeism, poor academic performance, and suicidal ideation [19,20,21,22]. Cyberbullying perpetration is also associated with anxiety, depression, low life satisfaction, loneliness and poor academic performance. Cyberbullying perpetrators are more likely to report drug and alcohol use, and violence, later in adult life [20,23,24].

### 1.3. Cyberbullying and Digital Piracy

Research that has examined the relationship between perpetrator and victim roles in aggressive behaviors, specifically in bullying processes, and digital piracy is relatively new. However, previous studies have pointed out how certain psychosocial variables are associated with deviant behaviors in cyberspace. For example, several researchers have demonstrated that having a positive attitude toward violent behavior is a significant predictor of other types of risk behaviors, such as digital piracy, and actual physical and verbal violence [25,26]. Previous research has also suggested that bullying, cyberbullying, and digital piracy may be predicted by deficits in moral values, moral emotions (lack of remorse), and/or morally disengaged justifications [4,27,28]. For cyberbullying, this finding can be explained by the absence of direct contact with the victim, which makes it easier for the bully to act immorally without feeling guilty and excuses him/herself from blame by denying any damage to or by deflecting blame on the victim [29,30]. The same process may be applied to digital piracy, where individuals are able to neutralize their wrongdoing by justifying their illegal actions as being “normal” or by applying cognitive strategies to dissociate themselves from moral responsibility [10]. Research should analyze if behaviors that share similar characteristics in cyberspace are associated by considering the different involvements; for example, according to the cyberbullying role played. To date, however, no studies have been conducted to examine the associations between involvement in cyberbullying and self-reported digital piracy among adolescents.

### 1.4. Theoretical Rationale

One potential explanation for the association between cyberbullying and digital piracy is the “generality of deviance” framework, which states that a wide range of antisocial behaviors positively correlate with one another because either one form of deviant behavior leads to involvement in other forms of deviance, or different forms of deviance have the same underlying causes [31,32,33]. This rationale is supported by substantial empirical evidence which demonstrates that the individuals who commit one form of deviance are likely to commit other forms as well; e.g., dangerous driving, problem gambling, illicit substance use, animal abuse, risky sexual behavior, or delinquency [31,33,34,35]. Ultimately, since various forms of deviant behavior tend to co-occur among individuals, adolescents who are involved in cyberbullying may also engage in digital piracy to a greater extent than those who are uninvolved in cyberbullying. 

### 1.5. Study Purpose

This study addresses the large gaps in the literature about the association between digital piracy and cyberbullying in adolescence. First, this study explores the specific association between self-reported digital piracy and cyberbullying roles (uninvolved, victims, bullies, and bully/victims). It is hypothesized that, for both sexes, adolescents who are bullies or bully/victims will be more likely to self-report digital piracy. This hypothesis is based on previous literature, which demonstrates that individuals who engage in a specific deviant behavior may also engage in other forms of deviant behavior.

Next this study analyses whether adolescents who are victims, bullies, or bully/victims (“cyberbullying roles”) differ in terms of the ethical consideration of ever accessing illegal context on the Internet. It is hypothesized that adolescents who are bullies and bullying/victims consider digital piracy a more ethical behavior that victims or uninvolved adolescents. This hypothesis may be supported by the fact that both cyberbullying perpetration and digital piracy are associated with moral disengagement. Thus, the view of digital piracy as being morally acceptable may be stronger among those individuals involved as perpetrators in other deviant behaviors.

## 2. Method

### 2.1. Study Design and Participants

The present study is a cross-sectional study in which 720 adolescents from grade 7 to grade 10 of compulsory secondary education in the Castile-La Mancha region (Spain) were invited to participate. Parental consent for participation was received from all but 26 participants, who were excluded from participating. Six hundred and ninety-four (96.3%) adolescents participated in the study and 643 completed the questionnaires without missing items. Of these 643 adolescents, 30% were from Grade 7, 28.5% were from Grade 8, 20.2% were from Grade 9, and 21.2% were from Grade 10. Gender composition was 49.3% females (*n* = 317) and 50.7% males (*n* = 326). Ages ranged from 13–18 years old (*M* = 14.56; *SD* = 1.45). Informed consent was obtained from all the individual participants included in the study. All the procedures performed in the study that involved human participants were in accordance with the ethical standards of the institutional and/or National Research Committee, and with the 1964 Declaration of Helsinki and its later amendments or comparable ethical standards.

### 2.2. Measurement Variables and Instruments

All the participants were administered a battery of tests to determine socio-demographic variables, such as age, gender, and grade level.

#### 2.2.1. Digital Piracy

Consistent with previous research on digital piracy [36,37], self-reported digital piracy was measured with an aggregate of five items that asked about the contents they have accessed illegally. On a five-point scale that ranged from very rarely (1) to very frequently (5), respondents marked how frequently they had downloaded or accessed streaming software, music, movies or TV series, books, and games without paying for them or using legal streaming platforms. Scores were obtained by summing items, where higher scores indicated a higher degree of self-reported digital piracy.

#### 2.2.2. Ethical Consideration of Digital Piracy

Based on previous literature [38,39], the ethical consideration of digital piracy was assessed by a single item asking participants to what extent they considered digital piracy to be an ethically and acceptable behavior on a five-point Likert scale that ranged from not acceptable (1) to completely acceptable (5).

#### 2.2.3. Cyberbullying Victimization and Perpetration

Cyberbullying experiences were devised by using the items from the Spanish Cyberbullying Questionnaire for measuring cyberbullying victimization (CBQ-V) [40] and cyberbullying perpetration (CBQ) [41]. Each used scale was a 10-item self-reported measure in which participants indicated how often they had been a victim of different behaviors on the Internet in the last three months. Items were scored on a five-point scale (1 = never, 2 = once or twice, 3 = two or three times a month, 4 = once a week, 5 = several times a week). An example item to measure cyberbullying perpetration was “writing embarrassing jokes, rumors, gossip, or comments about a classmate on the Internet”. The equivalent item to measure cyberbullying victimization was “writing embarrassing jokes, rumors, gossip, or comments about me on the Internet”. The internal consistency coefficient in this sample, measured through Cronbach’s Alpha, was 0.82 for the victimization scale, and 0.83 for the perpetration one.

#### 2.2.4. Additional Covariates

The covariates included in the analyses were gender, age, leisure activities and time spent on the Internet. Gender was operationalized as a dichotomous variable. Age was included as a continuous variable with values from 12 to 18 years. Time spent online and leisure activities were also included as continuous variables. Time spent online was measured following the procedure described by Meerkerk et al. [42]. Respondents were asked how many hours they spent online on a typical day when they used the Internet (five-point scale: from 5 h or more to less than 1 h). Regarding leisure activities, participants were asked to indicate how often they engaged in the following: (1) listening to music; (2) watching movies or TV series; (3) leisure reading; (4) playing video games. Answers were given on a five-point scale that ranged from 0 = never to 5 = every day.

### 2.3. Procedure

The study met the standards set by the Ethics Committee for research at the higher education institution. Adolescents completed the 35-min questionnaire in groups of approximately 25 students at school under the supervision of at least one of the researchers. Participants were assured that their individual responses would remain anonymous and would not be seen by their parents, peers, or teachers. Adolescents were informed that there were no right or wrong answers.

### 2.4. Analysis Plan

Descriptive statistics were examined for each study variable. Second, Pearson correlations were computed among all the study variables. Student’s *t*-tests were conducted to determine if there were significant gender differences in the prevalence of self-reported digital piracy and the ethical consideration of digital piracy. Multiple regression analyses were employed to determine the association between cyberbullying roles and self-reported digital piracy, by adjusting for age, leisure activities and time spent on the Internet, and by stratifying by gender. Finally, a covariance analysis (ANCOVA) was performed to ascertain the differences between cyberbullying roles in the ethical consideration of digital piracy using adolescents’ gender, time spent on the Internet, leisure activities and self-reported digital piracy as the covariables. SPSS 22.0 (IBM, Armonk, NY, USA) statistical software was used for all the analyses.

## 3. Results

### 3.1. Preliminary Analysis: Descriptive Analyses, Bivariate Correlations and Student’s t-Test

Table 1 presents the means, standard deviations and correlations for the measures used in the study. Correlations indicated that self-reported piracy is positively related with cyberbullying victimization and perpetration, time spent on the Internet, listening to music online, and watching movies or TV series online. Self-reported digital piracy was negatively related with playing video games. No significant relationship was found between leisure reading and self-reported digital piracy.

Regarding the means of these variables, statistically significant gender differences can be seen in Table 2. In terms of gender differences, males reported higher levels of self-reported digital behavior, cyberbullying perpetration, and playing video games. Males also considered digital piracy to be a more ethical and acceptable behavior than females did. Females’ means were significantly higher than those of males for listening to music, watching movies or TV series, leisure reading, and time spent on the Internet. No statistically significant differences for cyberbullying victimization were found between males and females.

### 3.2. Multiple Regression Analyses: Testing the Relationship between Cyberbullying Roles and Self-Reported Digital Piracy

Hierarchical multiple regression analyses were used to estimate the relationship of cyberbullying roles and self-reported digital piracy. Participants’ categorization as victims, bullies, or bully-victims of cyberbullying was done by following a highly restrictive criterion, similar to that used by other bullying researchers [43,44]. The students who indicated suffering, but not perpetrating, at least one of the behaviors included in the questionnaire several times a week were classified as victims. The students who reported perpetrating, but not suffering, at least one of the behaviors several times a week were classified as bullies. The students who indicated suffering and perpetrating at least one of the behaviors several times a week were classified as bully-victims. The remaining students were considered to not be involved in bullying. This procedure resulted in 41 (6.4%) adolescents categorized as cybervictims, 28 (4.4%) as cyberbullies, 25 (3.9%) as bully-victims, and 549 were uninvolved. Both the demographic variables (gender and ages) and leisure activities/time spent on the Internet were inputted into the regression analyses together with cyberbullying roles.

Multiple regressions were first computed for the whole sample (Table 3). The results indicated that the adolescents who reported listening to music, watching movies or TV series, and playing video games also reported higher digital piracy. In the same way, those who spent more time on the Internet also reported higher digital piracy. Among the cyberbullying roles, the adolescents who reported being bullies or bully/victims were much more likely to also report higher levels of digital piracy. No significant relationship was found between being a victim of cyberbullying and self-reported digital piracy.

Multiple regression analyses were conducted in the same way as they were above for males and females separately. The results (Table 4 and Table 5) indicated that the association between cyberbullying roles and self-reported digital piracy was more specific for one gender, particularly for males. Those who were bullies or bully/victims reported significantly more digital piracy. Moreover, the male adolescents who spent more time on the Internet reported greater digital piracy.

Among leisure activities, watching movies was associated with higher self-reported digital piracy for both genders. Listening to music was associated with only digital piracy for the male participants, whereas playing video games was associated with only digital piracy for the female participants.

### 3.3. ANCOVA Analysis: Examining Differences in the Ethical Consideration of Digital Piracy among Cyberbullying Roles

An ANCOVA was carried out to examine the differences in the ethical consideration of digital piracy among victims, bullies, bully-victims and uninvolved adolescents. As previously mentioned, we controlled for gender, time spent on the Internet, leisure activities, and self-reported digital piracy. The pair-wise differences in the adjusted means were examined using a post hoc test with Bonferroni correction.

The covariance analysis revealed the existence of statistically significant differences among the groups in the ethical consideration of digital piracy (see Table 6). The post hoc analyses indicated that cyberbullying bullies and bully-victims believed that digital piracy was a more acceptable and ethical behavior than what victims and the uninvolved group believed.

## 4. Discussion

This study examines the association between self-reported digital piracy and cyberbullying roles in a sample of 643 adolescents aged from 13 to 18 years old. The study aimed to extend the body of research on digital piracy by examining self-reported behaviors rather than attitudes or intentions, and by analyzing its relationships with other deviant behavior, specifically cyberbullying, after adjusting for other factors, such as age, leisure activities, and time spent on the Internet. This study also investigated the differences in the ethical consideration of digital piracy among cyberbullying victims, bullies, bully-victims, and uninvolved adolescents.

Regarding the association between self-reported digital piracy and cyberbullying roles, the results were in line with expectations. According to Hypothesis 1, involvement in cyberbullying as bullies and bully/victims increases the likelihood of engagement in digital piracy. Our results support the generality of deviance which suggests various forms of deviant behavior which, according to the study’s results, includes digital piracy, and tend to co-occur among individuals [34,35]. Digital piracy involves deliberately acquiring digital goods without paying [4]. Therefore, digital piracy is a similar intentional, and ultimately malicious act, to other behaviors, like cyberbullying perpetration. They also share similar characteristics; e.g., both behaviors involve little planning, are relatively easy to perform anonymously on any computer, and involve much fewer risks than traditional deviant behaviors. Thus, beyond intentionality and mischievous nature, both behaviors may share a certain overlapping nature because of their similar characteristics. The individuals who engage in a risk-taking domain, such as cyberbullying, may also be motivated to take risks more generally, including digital piracy. However, the association between cyberbullying perpetration and digital piracy only held for males when data were gender-stratified. Although more research is necessary, this difference between males and females could be related with previous studies which have shown that females get less involved in cyberbullying perpetration [45,46] and which also report less digital piracy than males [47]. This supports the gender socialization theory, which suggests that females and males have different perceptions and attitudes toward violence, crime, and ethical issues given their socialization differences [48,49,50].

Regarding the ethical consideration of digital piracy, according to Hypothesis 2, the results showed that after controlling for other factors, cyberbullies and cyberbully/victims considered digital piracy to be a more acceptable and ethical behavior than victims and uninvolved adolescents. The meta-analyses conducted by Lowry et al. [4] confirmed that potential pirates tended to have slightly stronger immoral views (i.e., piracy is acceptable) than moral views (i.e., piracy is unacceptable). The present study found that viewing digital piracy as an ethical behavior seems stronger among those involved in other deviant behaviors, specifically cyberbullying perpetration, who did not view piracy as being illegal or unethical. Thus, morality and immorality toward illegal acts may be directed by being involved in other deviant behaviors. This is in line with studies which have shown that tolerance and participation in non-normative behaviors are often justified by neutralization, including perpetrators’ denial of both responsibility and injury/harm/immorality [10]. According to the results of the present study, neutralization processes may be more salient among those involved in other immoral behaviors. This result confirms the importance of implementing prevention programs to address moral disengagement processes that involve rationalizations or justifications, which can increase the likelihood of getting involved in deviant behaviors, especially when addressing the possible co-occurrence of various forms of deviant behaviors. Future research should analyze the potential impact of moral competence (knowing what is right and wrong) and moral performance (what one actually does in the moral conflict context) over deviant behaviors, such as cyberbullying and digital piracy.

While the present study provides insight into the relationship between self-reported piracy and cyberbullying among adolescents, there are several limitations to the inferences that can be drawn from this work. One of our limitations is the use of self-reported measures. Although these instruments are an effective and reliable data collection method, the differences herein might be influenced by a response bias. Another limitation is that the sample consisted of secondary school students from a specific region of Spain. The associations between digital piracy and cyberbullying may differ in other samples. Future research should replicate this study with a more broadly representative sample. Another limitation lies in the nature of cross-sectional data collection. Due to the present study design, it was not possible to determine cause and effect. It is necessary to conduct future research that is better able to determine the direction of effects (i.e., longitudinal studies), and to replicate and validate these results by exploring a wider range of psychosocial variables (i.e., social norms, self-control, moral disengagement) because they may also contribute to the found pathways. Indeed another noteworthy limitation is that data pertaining to additional measures of digital piracy are lacking, such as hypothetical piracy vignettes. Additionally, we opted for a scale that ranged from very rarely (1) to very frequently (5) to indicate how frequently participants were involved in digital piracy. Consequently, participants are sort of forced to accept that there is some involvement in digital piracy. Future research must continue to understand how digital piracy and cyberbullying behaviors are related using experimentation rather than self-reporting surveys on piracy, and should also analyze different forms of digital piracy separately to know what factors are associated with each piracy type [4].

## 5. Conclusions

In summary, this study conducted with a sample of school-going non-clinical adolescents extends previous research by demonstrating that cyberbullying perpetration is associated with the consumption of digital goods without the copyright holder’s permission. Additionally, cyberbullying perpetrators viewed digital piracy as an ethically-acceptable behavior to a greater extent than victims and uninvolved youths in cyberbullying.

## Figures and Tables

**Table 1:** Correlations between the self-reported digital piracy, leisure activities, time spent on the Internet, and cyberbullying measures.

	M	SD	1	2	3	4	5	6	7	8	9
1. Self-reported digital piracy	2.53	1.09	-								
2. Ethical consideration of piracy	2.94	1.13	0.469 **	-							
3. Cyberbullying victimization	1.18	0.33	0.199 **	0.135 **	-						
4. Cyberbullying perpetration	1.15	0.36	0.252 **	0.224 **	0.537 **	-					
5. Listening to music	4.11	1.02	0.235 **	0.183 **	0.088 *	0.075	-				
6. Watching movies or TV series	2.87	1.02	0.338 **	0.165 **	0.054	0.011	0.325 **	-			
7. Leisure Reading	2.59	1.18	−0.076	0.158 **	−0.066	−0.112 **	0.080 *	0.082 *	-		
8. Playing video games	2.72	1.39	−0.265 **	0.138 **	0.169 **	0.121 **	−0.030	0.103 **	−0.194 **	-	
9. Time spent on the Internet	3.35	1.17	0.267 **	0.227 **	0.133 **	0.204 **	0.320 **	0.220 **	−0.148 **	0.171 **	-

* p < 0.05; ** *p* < 0.01.

**Table 2:** Gender differences in all the study variables.

	Females (*n* = 317)		Males (*n* = 326)		*t* (1, 643)	d
	M	SD	M	SD		
Self-reported digital piracy	2.35	1.02	2.70	1.14	−4.04 ***	0.32
Ethical consideration of piracy	2.84	1.06	3.03	1.19	−2.14 **	0.16
Cyberbullying victimization	1.16	0.30	1.19	0.36	−1.11	0.09
Cyberbullying perpetration	1.12	0.32	1.18	0.39	−2.24 **	0.16
Listening to music	4.34	0.89	3.89	1.09	5.72 ***	0.45
Watching movies or TV series	2.96	0.98	2.78	1.05	2.32 **	0.17
Leisure Reading	2.89	1.10	2.29	1.19	6.64 ***	0.52
Playing video games	1.98	1.07	3.45	1.27	−15.58 ***	1.25
Time spent on the Internet	3.45	1.20	3.26	1.14	2.10 **	0.16

*** *p* < 0.001; ** *p* < 0.01.

**Table 3:** Regression analyses: associations of leisure activities, time spent on the Internet, and cyberbullying roles on self-reported piracy behavior for the whole sample.

Dependent Measure	Self-Reported Piracy Behavior	
	b (S.E)	β
Gender	0.277 (0.095)	0.126 ***
Age	0.093 (0.036)	0.095 **
Listening to music	0.139 (0.042)	0.130 ***
Watching movies or TV series	0.280 (0.040)	0.261 ***
Leisure Reading	−0.024 (0.034)	−0.026
Playing video games	0.112 (0.034)	0.142 ***
Time spent on the Internet	0.106 (0.049)	0.090 *
Cyberbullying victim	0.262 (0.159)	0.058
Cyberbullying bully	0.546 (0.189)	0.101 ***
Cyberbullying bully/victim	0.498 (0.199)	0.088 **
F (df)	19.06 (10)	
R2 (∆R2)	0.25 (0.23)	

* *p* < 0.05; ** *p* < 0.01; *** *p* < 0.001.

**Table 4:** Regression analyses: associations of leisure activities, time spent on the Internet, and cyberbullying roles on self-reported piracy behavior for male participants.

Dependent Measure	Self-Reported Piracy Behavior	
	b (S.E)	β
Age	0.107 (0.054)	0.106 *
Listening to music	0.161 (0.056)	0.154 **
Watching movies or TV series	0.293 (0.057)	0.269 ***
Leisure Reading	−0.036 (0.053)	−0.035
Playing video games	0.088 (0.049)	0.098
Time spent on the Internet	0.171 (0.073)	0.138 *
Cyberbullying victim	0.148 (0.240)	0.031
Cyberbullying bully	0.639 (0.265)	0.121 **
Cyberbullying bully/victim	0.528 (0.258)	0.103 *
F (*df*)	9.98 (9)	
R2 (∆R2)	0.241 (0.217)	

* *p* < 0.05; ** *p* < 0.01; *** *p* < 0.001.

**Table 5:** Regression analyses: associations of leisure activities, time spent on the Internet, and cyberbullying roles on self-reported piracy behavior for female participants.

Dependent Measure	Self-Reported Piracy Behavior	
	b (S.E)	β
Age	0.075 (0.050)	0.080
Listening to music	0.107 (0.064)	0.094
Watching movies or TV series	0.276 (0.058)	0.267 ***
Leisure Reading	−0.011 (0.045)	−0.013
Playing video games	0.138 (0.049)	0.145 **
Time spent on the Internet	0.041 (0.068)	0.038
Cyberbullying victim	0.389 (0.213)	0.095
Cyberbullying bully	0.447 (0.275)	0.083
Cyberbullying bully/victim	0.585 (0.338)	0.090
F (*df*)	9.16 (9)	
R^2^ (∆R^2^)	0.231 (0.205)	

* *p* < 0.05; ** *p* < 0.01; *** *p* < 0.001.

**Table 6:** Summary of the ANCOVA results.

Variable	Cyberbullying Victims	Cyberbullying Bullies	Cyberbullying Bully/Victims	Uninvolved				
	M (SD)	M (SD)	M(SD)	M(SD)	Sum of squares	F	n2	R2 (∆R2)
Ethical consideration of digital piracy	3.47 a (1.13)	3.64 b (1.08)	3.78 b (1.09)	3.30 a (1.15)	316.40	3.31 **	0.019	0.357 (0.351)

Note: means that do not share subscripts differ at *p* < 0.05 in the multiple comparison Bonferroni procedure. Gender, self-reported piracy, and time spent on the Internet were controlled for. ** *p* < 0.01.
